# Stressing the Importance of CHOP in Liver Cancer

**DOI:** 10.1371/journal.pgen.1004045

**Published:** 2013-12-19

**Authors:** Barrett L. Updegraff, Kathryn A. O'Donnell

**Affiliations:** Department of Molecular Biology, UT Southwestern Medical Center, Dallas, Texas, United States of America; University of Washington, United States of America

Hepatocellular carcinoma (HCC) is the third leading cause of cancer death and the fifth most common solid tumor worldwide [1], [2]. Liver tumorigenesis is a multistep process in which external stimuli such as chronic inflammation or cirrhosis lead to the development of clonal populations of dysplastic hepatocytes that accumulate genetic changes and evolve into malignant foci [2]. Among the most common risk factors for HCC pathogenesis include viral hepatitis, alcoholism, and obesity [1], [3].

The same insults that predispose to HCC are known to induce endoplasmic reticulum (ER) stress pathways. One such pathway, known as the unfolded protein response (UPR), is triggered by the accumulation of incompletely folded proteins in the ER lumen [4]–[6]. Stimulation of the UPR results in the activation of three transmembrane proteins that induce downstream effectors to alter gene expression and ultimately modulate ER function. One of these UPR transmembrane proteins is protein kinase RNA (PKR)-like ER kinase (PERK), which phosphorylates eIF2α, leading to a transient translational blockade. A related pathway that shares transcriptional targets with the UPR is the integrated stress response (ISR) pathway. When triggered by viral infection or amino acid starvation the ISR also initiates eIF2α-dependent signaling events [7]. Although the UPR and ISR pathways are active in distinct human tumor types and the UPR is implicated in HCC [8]–[10], their relative contribution to the pathogenesis of HCC has remained uncharacterized.

In this issue of *PLOS Genetics*, Rutkowski and colleagues (DeZwaan-McCabe *et al.*, [11]) sought to determine whether the UPR pathway was induced in murine liver tumors that developed in a *Sleeping Beauty* (*SB*) transposon-induced insertional mutagenesis screen [12], [13]. The application of transposon-based approaches to cancer gene identification provides a powerful opportunity to examine the consequences of specific mutations in the context of *in vivo* tumor development [14]. Whole transcriptome sequencing of liver tumors generated in an *SB*-mediated liver tumorigenesis screen identified an induction of C/EBP Homologous Protein (CHOP), a stress-regulated transcription factor, in multiple *SB*-induced tumors. Upon further analysis, components of the two PERK-independent arms of the UPR pathway were not altered at the transcript level, leading the authors to further investigate the role of the ISR and CHOP in HCC.

CHOP, which has a diverse repertoire of transcriptional targets and modes of transcriptional modulation, was previously known to mediate apoptosis in response to ER stress [15]–[17]. Accordingly, several studies implicate CHOP as a putative tumor suppressor. In contrast to this, chromosomal translocations fusing *CHOP* to *FUS/TLS* and *EWS* have been identified in several cancers, hinting that CHOP may also play an oncogenic role in tumorigenesis in certain contexts [18], [19].

## The Integrated Stress Response in HCC: Not Just CHOPped Liver

**Figure 1:**
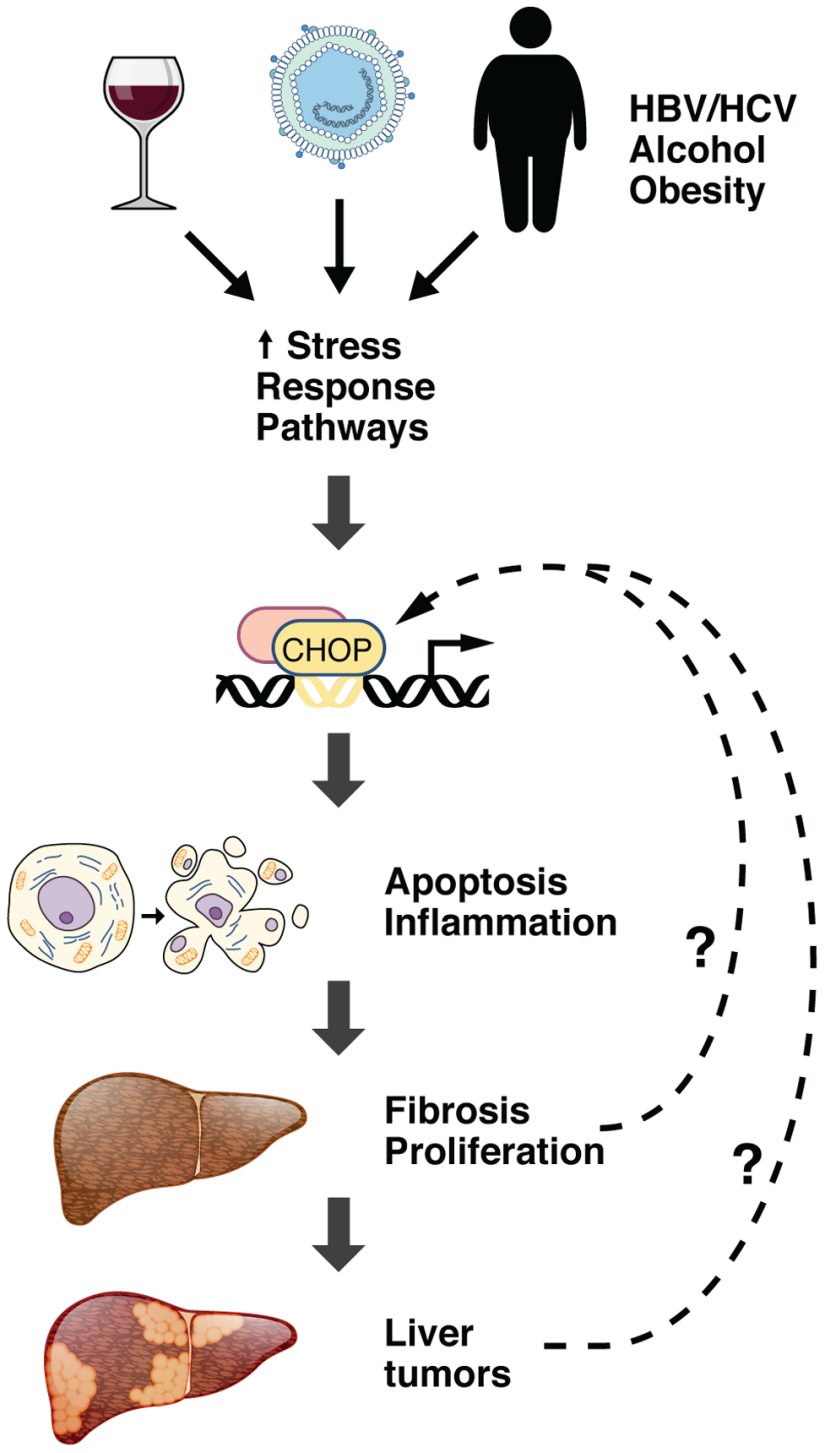
The role of CHOP in HCC pathogenesis. Hepatocyte injury, including toxicity from viral hepatitis, obesity, or alcoholism, promotes the induction of canonical ER stress pathways, including the UPR and ISR. This results in induction of the CHOP transcription factor, which stimulates cell death and provokes an inflammatory response. Inflammation may further stimulate fibrosis and compensatory proliferation, leading to the development of liver tumor nodules. It is also possible that stress caused by fibrosis and proliferation of dysplastic hepatocytes in HCC may induce expression of CHOP, further amplifying its effect on tumorigenesis.

Consistent with a pro-oncogenic role for CHOP, McCabe *et al.* [11] hypothesized that CHOP contributes to the pathogenesis of HCC *in vivo* by promoting apoptosis, inflammation, fibrosis, compensatory proliferation, and development of liver tumors (**Figure 1**). Consistent with this hypothesis, global deletion of *Chop* in mice attenuated these sequelae following treatment with the chemical carcinogen diethylnitrosamine (DEN). Following administration of the hepatotoxin carbon tetrachloride in wild-type mice, the authors observed an association of CHOP-positive foci with increased fibrosis. Staining of human HCC samples with a CHOP antibody revealed CHOP-positive foci in tumors and significantly less staining in normal liver. These results suggest that activation of CHOP promotes HCC progression. Moreover, these findings provide the first link between CHOP and liver oncogenesis.

Gene expression profiling of liver mRNA from *Chop*-null and wild-type mice in the absence of hepatotoxic challenge revealed that deletion of *Chop* reduced the levels of basal inflammatory signaling genes. This is consistent with an important role for CHOP in promoting inflammation after liver injury. Interestingly, genes encoding ribosomal proteins were significantly increased in liver tumors derived from DEN-treated *Chop*-null animals relative to tumors that developed in wild-type animals. None of these genes harbored canonical CHOP binding sites, leaving the question of how this occurs unresolved. This represents the first evidence that CHOP can reduce translation by suppressing expression of ribosomal proteins. However, this is consistent with the general role of the ISR as an inhibitor of translation. Further studies are needed to fully elucidate how CHOP affects the translational machinery and the resulting effects on translational output.

The authors of this study present several lines of evidence consistent with an oncogenic role for CHOP in promoting HCC. Their findings suggest that induction of CHOP is a common feature of liver cancer caused by viral infection, alcoholism, and obesity. Recently, a novel framework has been proposed suggesting that cancer cells exhibit hallmarks of chronic stress induced by DNA damage, proteotoxic stress created by accumulation of unfolded protein aggregates, metabolic stress, and oxidative stress [20]. Additional experiments are therefore warranted to determine whether CHOP induction is a causative event that promotes liver tumorigenesis and/or a consequence of the immense cellular stress that cells are subjected to as hepatocytes acquire mutations and undergo the multistep progression to HCC. This will require the generation of inducible and tissue-specific transgenic mouse models, which are currently lacking. Temporal manipulation of CHOP expression in the liver could also tease out whether CHOP promotes the initiation of HCC, or if it enhances tumorigenesis after dysplastic liver nodules form.

Given the resistance to HCC-associated phenotypes observed in *Chop*-null animals and the discovery of human HCC-associated CHOP expression, this stress-responsive transcription factor may serve as a useful biomarker for liver cancer. However, several important questions remain. For example, is CHOP-mediated apoptosis of hepatocytes the major initiating event that triggers the cycle of subsequent inflammation, fibrosis, and ultimately HCC initiation? Or does hepatocyte-specific expression of CHOP indirectly stimulate inflammation, perhaps through cytokine release, initiating the inflammation-tumorigenesis sequence? The analysis of CHOP target genes that mediate these effects in HCC will shed light on these issues. Perhaps most intriguingly, the identity of the eIF2α kinase that leads to CHOP induction in liver cancer remains unknown. PERK is one candidate, and it would be useful to determine whether PERK inhibitors will blunt CHOP expression and ameliorate HCC in mouse models. Thus, further investigation of the pro- and anti-oncogenic functions of CHOP is likely to reveal important new insights into the pathogenesis of liver cancer and other tumor types.
